# Expression and significance of cathepsin C and cathepsin D during pregnancy and Preeclampsia

**DOI:** 10.1186/s12958-023-01138-x

**Published:** 2023-10-04

**Authors:** Jingzhe Song, Nan Zhu, Xinchen Pan, Lu Guo, Xiang Kong

**Affiliations:** 1 Department of Obstetrics and Gynecology, Clinical Medical College, Yangzhou University, 225001 Yangzhou, Jiangsu China; 2 Graduate School of Dalian Medical University, Dalian, China; 3 School of Nursing and School of Public Health, Yangzhou University, Yangzhou, China; 4 Institute of Translational Medicine, Medical College, Yangzhou University, Yangzhou, Jiangsu Province China; 5 Jiangsu Key Laboratory of Experimental & Translational Non-Coding RNA Research, Yangzhou, Jiangsu Province China

**Keywords:** Cathepsin C, Cathepsin D, Pregnancy, Preeclampsia

## Abstract

**Background:**

Cathepsin C (Cat C) is involved in the inflammatory-immune system and can be degraded by cathepsin D (Cat D). Preeclampsia (PE) and the inflammation-immunity relationship is currently a hot research topic, but there are still few studies. The aim was to investigate the expression and significance of Cat C and D in the serum of nonpregnant women, patients in various stages of pregnancy and patients with PE, and in the placenta of patients with normal pregnancy and PE.

**Methods:**

Sixty young healthy nonpregnant women were selected: 180 normal pregnant women, including 60 each in the first, second, and third trimesters, and 100 women with PE, including 39 women with severe preeclampsia. The levels of Cat C and D in serum were detected by enzyme-linked immunosorbent assay (ELISA), and the expression levels of Cat C and D in placentas were detected by immunohistochemistry (IHC).

**Results:**

The serum of Cat C in the first trimester was significantly lower than that in the nonpregnant group (*P* < 0.001), whereas Cat D was significantly higher than that in the nonpregnant group (*P* < 0.01). The levels of Cat C and D in the second trimester and third trimester were significantly higher than those in the first trimester (*P* < 0.05), but there was no significant difference in Cat C and D between the second trimester and third trimester. The levels of Cat C in the serum and placentas of patients with PE were significantly higher than those in the third trimester (*P* < 0.001) and positively correlated with the severity of PE (*P* < 0.001), whereas the levels of Cat D in the serum and placentas of patients with PE were significantly lower than those in the third trimester (*P* < 0.001) and negatively correlated with the severity of PE (*P* < 0.001). Age, primigravida proportion, and body mass index were significantly higher in the PE group than in the control group (*P* < 0.05), which were high-risk factors for PE.

**Conclusions:**

Cat C and D are associated with the maintenance of normal pregnancy. In patients with preeclampsia, a significant increase in Cat C and a significant decrease in Cat D levels may lead to the occurrence and development of preeclampsia.

**Cathepsin C and cathepsin D are risk factors for preeclampsia: a case control study.**

This study provides for the first time the changes in serum and placental levels of cathepsin C and cathepsin D in nonpregnant subjects, in all stages of pregnancy, and in patients with preeclampsia at the same time, and explores their significance. We found that the serum cathepsin C expression is significantly lower in the first trimester than in the nonpregnant control, second trimester, and third trimester; cathepsin C expression is significantly higher in the serum and placenta of patients in the preeclampsia group than in the third trimester, and is consistent with disease severity. The expression of serum cathepsin D gradually and significantly increases in the nonpregnant controls, early pregnancy and mid-gestation; the serum cathepsin C and D do not change in the second trimester and third trimester. The expression of cathepsin D is significantly lower in serum and placenta of patients in the preeclampsia group than in the third trimester. We also verified that age, proportion of primigravida and body mass index are significantly higher in the preeclampsia group than in the control group, which are high-risk factors for preeclampsia.

## Background

Human pregnancy is a complex and delicate process, from the implantation of blastocysts to the formation of the placenta and the development of the placenta and fetus, requiring trophoblast cells to constantly invade the maternal endometrium, which is similar to the invasive behavior of tumor cells. However, it is very subtly regulated, making this invasive behavior restricted to the uterus. It has been shown that the invasive behavior of trophoblast cells during pregnancy is dependent on a large number of protein hydrolases, especially matrix metalloproteinases (MMPs), cathepsins, and serine proteases [1, 2].

Preeclampsia (PE), a pregnancy-specific condition, is a substantial contributor to maternal and perinatal morbidity and mortality as well as a major cause of iatrogenic premature birth, affecting 5–8% of pregnant women annually [3]. The pathogenesis and etiology of PE have not been fully addressed over the years, and the widely recognized explanations, such as placental ischemia, inflammatory immunological hyperactivation, and endothelial dysfunction, are insufficient to properly explain the precise etiology of PE. The theory of placental ischemia suggests that pathological placental ischemia results from incomplete invasion of trophoblast cells into the endometrium and impaired recasting of placental vasculature during gestation. This pathological placenta releases several inflammatory factors that damage the maternal vascular endothelium, resulting in maternal dysfunction of multiple organ systems such as hypertension, proteinuria, and fundus damage, among other clinical symptoms of PE [3, 4]. The initial cause of placental ischemia in PE is the decreased capacity of trophoblast cells to invade the endometrium and spiral arteries, and cathepsin is an important hydrolase that affects the invasion ability of trophoblast cells [5]. Therefore, this suggests that alterations in cathepsin expression and activity may be a key connection in the etiology of PE.

Cathepsins are protein hydrolases that can be classified as serine proteases, cysteine proteases, and aspartic proteases. Humans have been found to contain 15 different cathepsin types, including cathepsins A, B, C, D, E, F, G, H, K, L, O, S, V, W, and Z. These enzymes are active in lysosomal environments at an acidic pH, and are essential for normal physiological functions such as inflammatory signaling pathways, angiogenesis, and innate immunity, among many other processes [6, 7].

Cathepsin C (Cat C) is a cysteine protease that exists in diverse human tissues and cells, and its distribution in the placenta is mainly located in the syncytiotrophoblast, leukocytes, and specific areas of the maternal surface, but not in the cytotrophoblast. It has been demonstrated that Cat C expression is significantly higher in nondecidualized extravillous trophoblast cells than in decidualized extravillous trophoblast cells, and impaired uterine decidua has been reported in women with PE [8]. Cat C not only activates the p38 mitogen-activated protein kinase (p38MAPK) pathway to affect the degree of placental implantation, but also activates the nuclear factor-kappaB (NF-κB) pathway to cause maternal production of a series of inflammatory factors, leading to vasospasm and vascular endothelial damage, indicating that Cat C is crucial for both pregnancy and the pathophysiology of PE [9–12]. Cathepsin D (Cat D) is an aspartic protease that can be found in all cells, tissues, and organs except mature lysosome-free erythrocytes. Cat D has been reported to have proinflammatory and regulatory angiogenic effects and to be associated with invasion and phagocytosis of rodent trophoblast cells, which can affect uterine spiral artery recasting and placental implantation, suggesting that Cat D is also essential for normal pregnancy and the cause of PE [5, 13–16].

PE is closely related to inflammation-immunity, which is the focus of current research. Cat C and D have been reported in pregnancy and preeclampsia studies, but the results are inconclusive [9–17]. There has not been a simultaneous study of nonpregnant, normal pregnant, and preeclamptic patients. This study explored the changes in these two factors in nonpregnant women, pregnant women, and women with preeclampsia to understand the possible relationship with the development of preeclampsia.

## Materials and methods

### Research subjects

Women attending Northern Jiangsu People’s Hospital from November 2021 to May 2023 were selected. Among them were 60 young healthy nonpregnant women, 180 normal pregnant women, including 60 each in the first, second, and third trimesters, and 100 PE, of which 39 were severe. Criteria for diagnosing preeclampsia [18]: new-onset hypertension (systolic blood pressure maintained at ≥ 140 mmHg and/or diastolic blood pressure maintained at ≥ 90 mmHg) with proteinuria and/or end-organ dysfunction after 20 weeks of gestation. None of the study subjects received a blood transfusion or immunotherapy before specimen collection, and the exclusion criteria included cases of smoking, chronic hypertension complicated by preeclampsia, HELLP syndrome, pregnancy complicating chronic hypertension, diabetes mellitus, renal illness, or autoimmune disease.

### Enzyme-linked immunosorbent assay

The nonpregnant group was chosen at random from the local healthy female population, and the rest of the study subjects were sampled by drawing 5 ml of venous blood from the elbow in a test tube before outpatient or inpatient intravenous treatment. The specimen was centrifuged at 3500 r/min for 15 min at room temperature after standing for 30 min, and the supernatant was collected and kept at -80 °C until analysis. According to the manufacturer’s protocol, serum Cat C and D levels were measured using Cat C and D ELISA kits (HB2902Hu, HS2902-Hu; HB022-Hu, HS022-Hu; Shanghai Hengyuan Biotechnology LTD), and final results were expressed as pg/mL.

### Immunohistochemistry

Twenty cases were selected from each group in the third trimester, preeclampsia alone and severe preeclampsia. Their placentas were collected immediately after delivery, avoiding calcification and vascularization, and the central maternal surface of the placentas was retained at a size of approximately 1 cm*1 cm*1 cm. The placentas were soaked in 10% formalin for 24 h, dehydrated, and paraffin-embedded until use. Graded ethanol and xylene were used to hydrate and dewax paraffin sections. To execute antigen repair, the samples were placed in a repair cassette with EDTA (pH 9.0) antigen repair buffer. The sections were washed in PBS (pH 7.4) buffer after cooling naturally. To inhibit endogenous peroxidase, sections were treated in a 3% peroxide solution at room temperature for 25 min without exposure to light. The sections were sealed at room temperature for 30 min after being washed once more and covered with 3% BSA dropwise. The primary antibody (cathepsin C antibody: TA371282S, cathepsin D antibody: TA368769S, Origene, USA) was incubated with the sections overnight at 4 °C in a wet box. The secondary antibody (HRP-goat anti-rabbit, RCA054, Shanghai Rutron Biotechnology LTD.) was then added after washing, and it was incubated for an hour at room temperature. Following washing, a drop of DAB color developer was added at a time. Finally, the nuclei were restained, dehydrated and sealed. A Nikon Eclipse CI-S microscope and a Nikon DS-U3 image system (Nikon, Japan) were used to take the pictures.

The staining pattern was observed by two research personnel who were unaware of the experimental conditions, indicating immunoreactivity. A composite score was given in terms of both the percentage of positive cells and staining intensity. Semiquantitative analysis was performed to estimate the intensity of sample staining: 0 indicates no reaction, 1 indicates the presence of both negative and trace positive cells, 2 indicates moderate staining, and 3 indicates strong staining. The presence of brownish-yellow or yellowish-brown particles in the cytoplasm was judged as positive: grades 0 and 1 were determined by the percentage of positive cells that were less than 5%, between 5% and 30%, between 30% and 60%, and greater than 60%. Grade 3 was determined by the percentage of positive cells that were greater than 60%. By multiplying the two values, one can obtain the final score, where 0 represents a negative (-), 1 to 2 a moderately positive (+), 3 to 4 a positive (++), and 6 to 9 a very positive (+++). Five fields of view were chosen for each part, and one portion was chosen for each specimen.

Before sample collection, all subjects signed written informed consent forms. The Subei People’s Hospital Medical Ethics Committee approved the sample collection and use.

### Statistical analysis

Statistical analysis was performed using R version 3.5.3 software. Measurement information is presented as the mean ± standard deviation or median (interquartile spacing), and comparisons of two samples were made using the T test, while comparisons of multiple samples were made using the Kruskal‒Wallis test. The correlations were examined using Spearman rank correlation, and the count data were subjected to the R×C table χ2 test. The difference was shown to be statistically significant at *P* < 0.05.

## Results

### General situations

Table 1 displays the clinical traits of the study participants. Age, body mass index (BMI), gestational week, primigravida and blood pressure were not significantly different between nonpregnant and first trimester pregnancies (*P* > 0.05). Age, primigravida, and blood pressure were not statistically significant (*P* > 0.05), and BMI and gestational week were significantly different (*P* < 0.001) between normal trimesters. Age, BMI, gestational week, primigravida, and blood pressure were statistically significant in PE compared with the third trimester (*P* < 0.05).

**Table 1:** Clinical information of subjects in the study

Variables	Nonpregnant	First trimester	Second trimester	Third trimester	Preeclampsia	*P value*
Age (years)	28.6 ± 2.92	28.85 ± 2.97	29 ± 2.95	28.9 ± 2.98	30.74 ± 3.51	0.002^c^
BMI (kg/m2)	21.7 ± 2.5	21.93 ± 2.51	24.55 ± 2.75^a^	27.16 ± 2.73^b^	29.76 ± 4.07	0.000^c^
Gestational week for serum collection (week)	-	8.81 ± 2.58	21.28 ± 3.72^a^	39.68 ± 0.94^b^	36.52 ± 2.78	0.000^c^
Gestational week of delivery(week)	-	-	-	39.82 ± 0.96	36.77 ± 2.42	0.000^c^
Primigravida	41/60	38/60	37/60	36/60	78/100	0.015^c^
Systolic blood pressure (mmHg)	115.1 ± 7.74	112.15 ± 7.90	112.20 ± 8.05	113.2 ± 7.88	157.22 ± 10.34	0.000^c^
Diastolic blood pressure (mmHg)	73.95 ± 3.48	73.4 ± 3.24	73.5 ± 3.24	72.05 ± 3.52	105.54 ± 8.0	0.000^c^

BMI: body mass index

Data are expressed as the mean ± SD

^a^ Second trimester compared with first trimester: *P* < 0.001; ^b^ third trimester compared with second trimester: *P* < 0.001;^c^ preeclampsia compared with third trimester: *P* < 0.05

### Expression of cat C and D in the serum of nonpregnant women, women at all stages of pregnancy, and women with PE

**Fig. 1:**
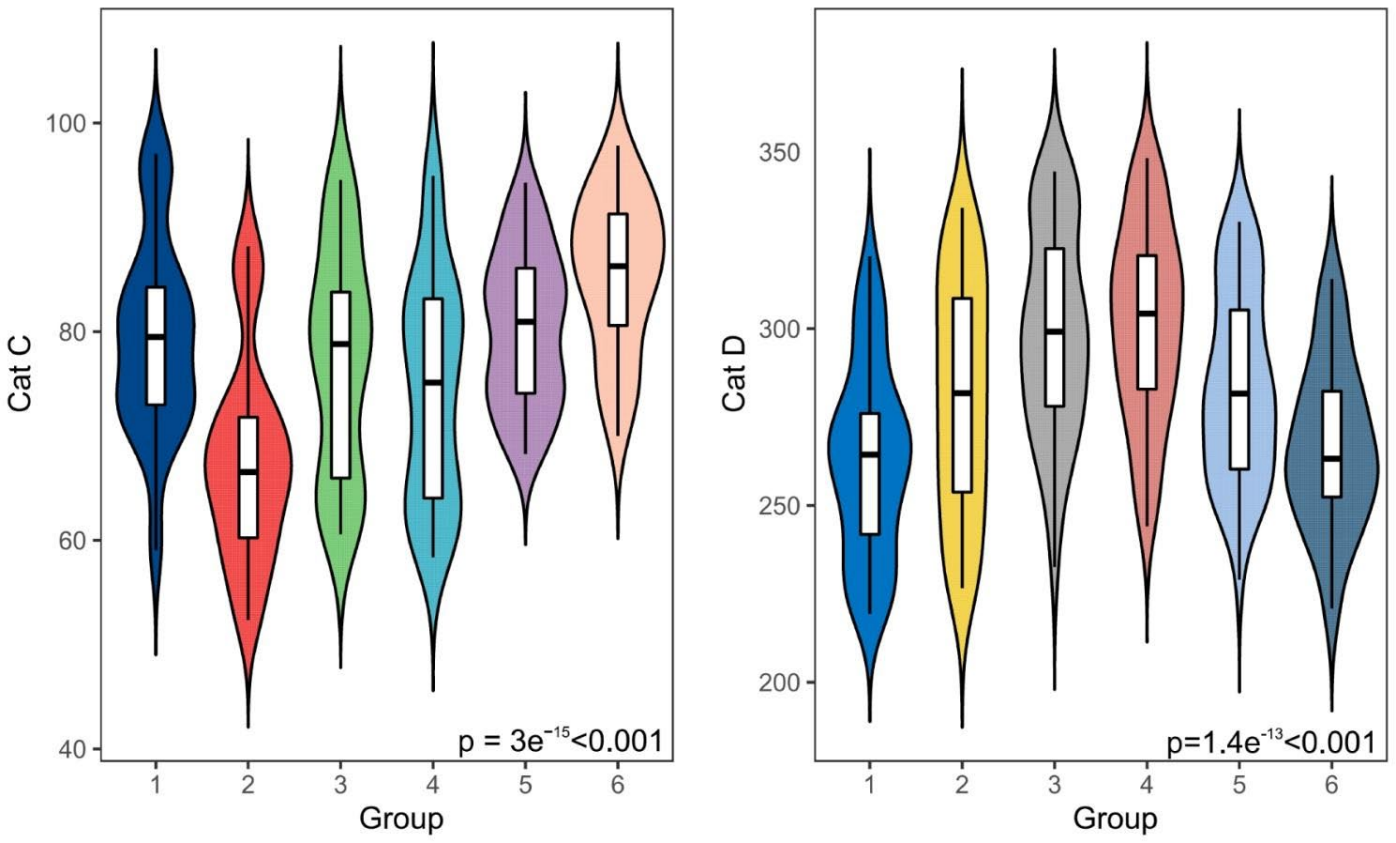
Serum expression of Cat C and D in the study groups. 1: nonpregnant; 2: first trimester; 3: second trimester; 4: third trimester; 5: preeclampsia alone; 6: severe preeclampsia

Serum Cat C expression was significantly lower in the first trimester than in the nonpregnant control, second trimester, and third trimester (*P* < 0.001; *P* < 0.001; *P* = 0.01 < 0.05), whereas the difference in Cat C in the nonpregnant control, second trimester, and third trimester was not statistically significant. The expression of Cat C in the serum of PE was significantly higher than that in the third trimester (*P* < 0.001). Cat C was significantly different in a two-by-two comparison between the three groups of the third trimester, preeclampsia alone, and severe preeclampsia (*P* < 0.05) (Table 2; Fig. 1).

The expression of serum Cat D gradually increased in the nonpregnant control, first trimester, and second trimester, with a statistically significant difference (*P* < 0.05). Cat D was not significantly different in the second trimester and third trimester. The expression of Cat D in the serum of PE was significantly lower than that in the third trimester (*P* < 0.001). Cat D was significantly different in a two-by-two comparison between the three groups of the third trimester, preeclampsia alone, and severe preeclampsia (*P* < 0.05) (Table 2; Fig. 1).

**Table 2:** Expression of Cat C and D in serum of nonpregnant, all stages of pregnancy and PE

Biomarker	Nonpregnant(*n* = 60)	First trimester (*n* = 60)	Second trimester (*n* = 60)	Third trimester (*n* = 60)	Preeclampsia Alone (*n* = 61)	Severe preeclampsia (*n* = 39)	*P*
Cat C (pg/mL)	79.481 (73.0,84.2)	66.537 (60.0,71.9)	78.804 (65.6,85.1)	75.100 (63.8,83.3)	80.934 (74.0,86.2)	86.264 (80.2,91.4)	0.000^a^ 0.000^b^ 0.010^c^ 0.453^d^ 0.020^e^ 0.000^f^ 0.017^g^
Cat D (pg/mL)	264.372 (240.9,276.5)	281.711 (253.7,309.3)	299.177 (277.5,327.2)	304.284 (280.9,321.2)	281.625 (259.3,305.8)	263.227 (251.0,282.4)	0.008^a^ 0.015^b^ 0.004^c^ 0.921^d^ 0.006^e^ 0.000^f^ 0.020^g^

Data are expressed as Median (P25, P75)

^a^Non-pregnant compared with first trimester; ^b^first trimester compared with second trimester; ^c^first trimester compared with third trimester; ^d^second trimester compared with third trimester; ^e^preeclampsia alone compared with third trimester; ^f^severe preeclampsia compared with third trimester; ^g^severe preeclampsia compared with preeclampsia alone

### Expression of cat C and D in normal pregnancy and PE placentas

**Fig. 2:**
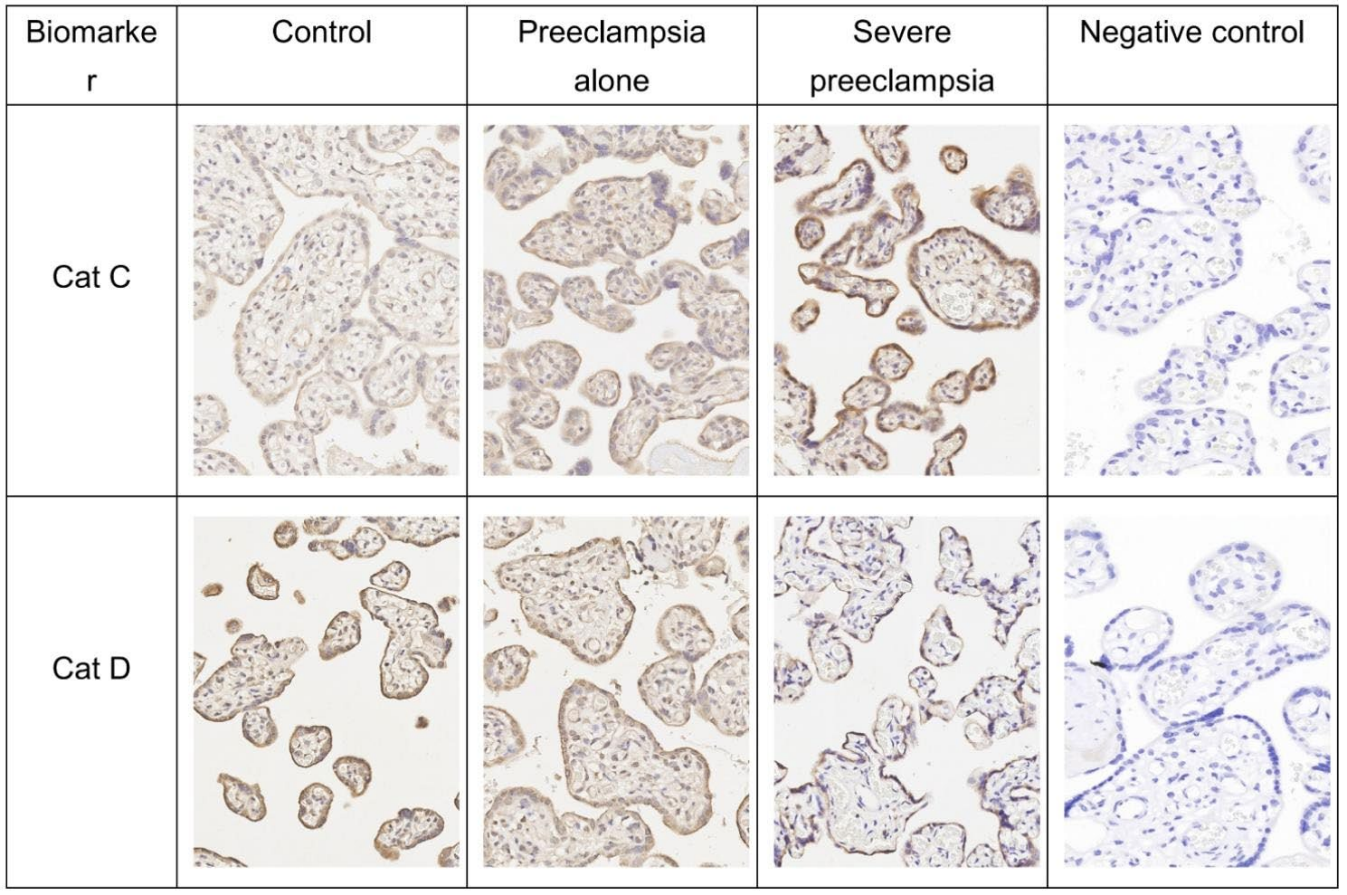
Cat C and Cat D expression in normal pregnancy and PE placentas. Picture magnification ×200

Cat C was expressed predominantly in the trophoblast layer in PE placentas, and Cat D was expressed predominantly in the trophoblast layer in normal pregnancy placentas (Fig. 2). The expression of Cat C in placental tissue was significantly higher in PE than in normal pregnancy (*P* < 0.001), and the expression of Cat C was significantly increased in the preeclampsia alone and severe preeclampsia groups compared with the control group (χ2 = 20.746, *P* < 0.001; χ2 = 66.546, *P* < 0.001). The expression levels of Cat C were also significantly increased in severe preeclampsia compared with preeclampsia alone (χ2 = 25.031, *P* < 0.001), and all these differences were statistically significant (Table 3). The expression of Cat D in placental tissue was significantly lower in PE than in normal pregnancy (*P* < 0.001); among them, the expression of Cat D was significantly lower in the preeclampsia alone and severe preeclampsia groups than in the control group (χ2 = 16.314, *P* = 0.001 < 0.01; χ2 = 54.299, *P* < 0.001). Cat D expression levels were also significantly lower in severe preeclampsia than in preeclampsia alone (χ2 = 13.643, *P* = 0.003 < 0.01); all differences were statistically significant (Table 3).

### Correlation of cat C and D expression in serum and placentas with PE disease severity

We found that Cat C in serum and placentas was significantly and positively associated with the severity of PE disease (*r* = 0.422, *P* < 0.001; *r* = 0.489, *P* < 0.001), whereas Cat D in serum and placentas was significantly and negatively associated with the severity of PE disease (*r*=-0.450, *P* < 0.001; *r*=-0.403, *P* < 0.001) (Table 4).

## Discussion

Normal pregnancy requires normal placental development, and thorough differentiation and invasion of trophoblast cells are necessary for placental development. The extracellular matrix is degraded by cathepsins in this invasive action. During pregnancy, the maternal immune system is regulated by a series of strict and precise immune balances in pro- and anti-inflammatory processes. Excessive proinflammatory processes or decreased trophoblast invasive capacity can cause several serious pregnancy complications, such as PE. However, the etiology of PE is currently unknown. Therefore, in this study, we determined the changes in Cat C and D in the serum of preconception, all stages of pregnancy and PE, as well as the expression in the placenta of normal pregnancy and PE, and explored the potential mechanisms of Cat C and D in embryo implantation, placenta formation, and the maternal immune system.

Cat C, commonly known as dipeptidyl peptidase I, is a lysosomal exopeptidase that is a member of the papain superfamily. The activation of pro-inflammatory granule-associated serine proteases is Cat C’s most emblematic physiological function. Once activated, these proinflammatory proteins can breakdown different extracellular matrix substances, resulting in tissue damage and persistent inflammation [19]. Cat C is currently drawing more attention due to its biological significance in many systemic disorders, including rheumatoid arthritis, periapical periodontitis, and malignancies [20–22]. However, there has not been much discussion of the precise involvement of Cat C in the development of PE and pregnancy. Ellen et al. [8] reported that Cat C expression is significantly lower in decidualized extravillous trophoblast cells than in nondecidualized extravillous trophoblast cells. This finding was exactly consistent with the trend of low expression of Cat C in normal early pregnancy serum in this study. Cat C activates immunity and drives neutrophils to the site of infection. Therefore, this study found that elevated serum Cat C levels in mid- and late pregnancy return to prepregnancy levels and may be associated with the maintenance of normal immunity in the organism.

PE begins with placental ischemia due to decreased trophoblast invasive capacity, and placental ischemia can further cause maternal inflammatory-immune dysregulation. It has been documented that women with PE have impaired uterine decidua [23]; hypoxia/reoxygenation injury promotes Cat C expression and activation of the p38MAPK/NF-κB pathway in human umbilical vein endothelial cells, but downregulation of Cat C inhibits p38MAPK/NF-κB pathway activation and increases vascular endothelial cell activity, such as enhancing endothelial cell invasion, anti-apoptosis, antioxidant capacity and angiogenic potential [24]. The p38MAPK signaling pathway regulates apoptosis and oxidative stress in endothelial cells in PE and affects the angiogenic activity of extravillous trophoblasts; hyperactivation of the p38MAPK pathway can lead to a variety of diseases, such as PE [9, 10]. As a classical inflammatory and oxidative stress signaling pathway, the NF-κB pathway can lead to endothelial stress, uteroplacental dysfunction, and PE [11, 12]. Another study showed that the expression of Cat C and chymase is significantly increased in peripheral vascular endothelial cells of PE patients compared with normal pregnancy controls, and upregulation of Cat C induces chymase activation in endothelial cells [25]. Chymase not only converts angiotensin I to angiotensin II but also converts macromolecule endothelin to endothelin-1, which produces downstream vasoconstriction through angiotensin II and endothelin and has a crucial role in the regulation of vascular function. In this study, Cat C expression in serum and placenta was considerably greater in PE compared to normal pregnancy, consistent with the severity of the disease. Therefore, we hypothesize that Cat C may be involved in the whole process of PE by affecting uterine decidua, interfering with placental attachment, and causing a maternal inflammatory response and vascular dysfunction.

**Table 3:** Expression of Cat C and D in normal pregnancy and PE placentas

Biomarker	Group	Number of cases	Number of views	-	+	++	+++	χ^2^	*P*
Cat C	Normal pregnancy	20	100	45	37	12	6	20.746	0.000^a^
	Preeclampsia alone	20	100	18	41	27	14	66.546	0.000^b^
	Severe preeclampsia	20	100	8	19	33	40	25.031	0.000^c^
Cat D	Normal pregnancy	20	100	3	10	35	52	16.314	0.001^a^
	Preeclampsia alone	20	100	11	26	30	33	54.299	0.000^b^
	Severe preeclampsia	20	100	19	44	19	18	13.643	0.003^c^

^a^ Preeclampsia alone compared with normal pregnancy: *P* < 0.01;^b^ severe preeclampsia compared with normal pregnancy: *P* < 0.001;^c^ severe preeclampsia compared with Preeclampsia alone: *P <* 0.01

Cat D is an endopeptidase within the lysosome and is usually involved in protein degradation within the lysosome and endosomal cells, as well as in the processing of precursor proteins. Cat D has optimal in vitro activity at pH 3.5–5.5 when the extracellular matrix is the substrate [5, 26]. In vitro, studies have shown that Cat D degrades Cat C and activates other zymogens, promoting trophoblast cell invasion during implantation and placenta formation [13, 27]. A mouse experiment showed that the expression and presence of Cat D in the extracellular space at the maternal-fetal interface is positively correlated with trophoblast invasiveness and suggests that invasive trophoblast cells are involved in Cat D release [28]. In normal pregnancy, there are two peaks of trophoblastic cell invasion of the uterus, one occurring during the 10th week of gestation and the second during the 14th to 20th week of gestation [29]. Thus, we found a significant increase in serum Cat D expression in the first trimester compared to the nonpregnant group and a further significant increase in the second trimester compared to the first trimester, which may be associated with placental trophoblast invasion. Cat D can activate MMPs directly or indirectly [30]. MMPs degrade the extracellular matrix and are involved in vascular remodeling. One study found that circulating levels of MMPs increase significantly and remain high during pregnancy [31]. Cat D expression in the serum of this study was maintained at high levels during the third trimester, which may be connected with analogous physiologically high levels of MMPs during pregnancy. Kim et al. [5] reported that the levels of Cat D in the first trimester were significantly lower than those in nonpregnant women, but the levels of Cat D in the third trimester were considerably greater than those in the first trimester, which is consistent with our findings.

**Table 4:** Correlation of Cat C and D expression in serum and placentas with PE disease severity

Biomarker	serum	placentas
Cat C, *r* *P* value	0.422	0.489
	0.000***	0.000***
Cat D, *r* *P* value	-0.450	-0.403
	0.000***	0.000***

* *P* < 0.05 ** *P* < 0.01 *** *P* < 0.001

Reports on the correlation between Cat D and PE are limited, and the results vary. One study found overexpression of Cat D in the placenta of PE patients, which is thought to be associated with triggering apoptosis and reducing reactive oxygen species scavenging [15]. Nakajima et al. [14] reported that Cat D activity is higher in PE serum than in normal pregnant women, and activated Cat D has an antiangiogenic effect by cutting prolactin, growth hormone, and placental lactogenic hormone and generating vasopressors. However, it should be noted that under physiological pH conditions, the production of vasopressor by cleavage of prolactin by Cat D does not occur in human serum, and under acidic pH conditions, only limited vasopressor is produced by cleavage of prolactin in serum; therefore, the production of vasopressor requires the involvement of the local cellular and tissue microenvironment [32]. Another study showed that prolactin fragments with antiangiogenic effects are highly expressed in the placentas of gestational hypertension, whereas the expression of Cat D in the placentas of the gestational hypertension group is significantly decreased [33]. Kim et al. [16, 34] found low expression of Cat D in PE serum, and it is believed that insufficient Cat D expression or activity reduces the invasiveness of trophoblast cells. Nakashima et al. [35] also found significantly low expression of Cat D in PE placentas through experiments, and further tests proved that hypoxia can inhibit the expression of Cat D in trophoblast cells. In this study, Cat D was found to be expressed at low levels in the PE placenta and serum and inversely correlated with disease severity. Therefore, we hypothesize that the involvement of Cat D in the initiation of PE may be related to decreased activation of MMPs, resulting in inadequate reconstruction of uterine spiral arteries and superficial invasion of placental trophoblasts.

The major weaknesses of our study are our cross-sectional design and lack of prospective studies. Second, our study requires further testing of enzyme activity and detection of enzyme expression in chorionic villi during early pregnancy to refine the actual effects of cathepsin in pregnancy and PE.

## Conclusions

We found that low expression of Cat C and high expression of Cat D in serum in the first trimester facilitates pregnancy formation. Cat C and D are significantly elevated in the second and third trimesters, facilitating pregnancy maintenance and immune homeostasis. It has been found that these two factors are involved in normal pregnancy, but an imbalance may be involved in the development of PE. Therefore, continued research by knocking out the Cat C gene and activating the Cat D gene may offer a novel avenue for PE treatment.

## Data Availability

The datasets used and/or analysed during the current study are available from the corresponding author on reasonable request.
